# Supplementary material for: The nociceptive response induced by different classes of Tityus serrulatus neurotoxins: The important role of Ts5 in venom-induced nociception
Source: PLoS Negl Trop Dis. 2023 Jan 30;17(1):e0011057. doi: 10.1371/journal.pntd.0011057 (PMC9886245; doi:10.1371/journal.pntd.0011057)
Supplement: Attachment — Submitted filename: Response_to_reviewers_MBP.docx [file pntd.0011057.s002.docx]

***Reviewer 1:***

*Specific points that need the authors' attention:*

*1.- Spontaneous nociception?? As pain cannot be directly measured in rodents, many methods that quantify “pain-like” behaviors or nociception have been developed. These behavioral methods can be divided into stimulus-evoked or non-stimulus evoked (spontaneous) nociception, based on whether or not application of an external stimulus is used to elicit a withdrawal response. Authors induced a pain response by injecting Ts venom or isolated Ts neurotoxins. As far as I understand this action should be regarded as stimulus-evoked nociception rather than spontaneous nociception...*

Response: We understand the observation of the referee. However, in neuroscience, the measurement of pain can be divided into stimulus-evoked or non-stimulus evoked (spontaneous) nociception, based on whether or not application of an external stimulus is used to elicit a withdrawal response. Thus, the classification referred to the method and not to the noxious agent (*e.g.* toxin/drug) used to initiate pain. Stimulus-evoked methods, which include manual and electronic von Frey, Randall-Selitto and the Hargreaves test, which uses mechanical, heat, and cold stimuli. Thus, the classification of the method used is correct.

*2.- Toxin concentrations were calculated using ProtParam, an algorith accessible in the Expasy server. Theoretical extinction coefficients calculated from the amino acid sequence of a protein were developed before the turn of the (XXI) century and are poorly known by most researchers of the omics era. It is thus relevant to cite original reference(s) and briefly describe, in the corresponding manuscript's section, the methodology underlying the calculation.*

**Response:** Indeed, there are several methods for measuring protein concentration. One of the most used is absorbance analysis at 280 nm. None of the methods are 100% effective and corresponds to 100% of the amount obtained. Thus, the extinction coefficient is an important measure absorbance to correct this expected error between the different proteins’ measurements analyzed. The extinction coefficient, molar absorptivity or ε (epsilon) is a measure of the rate of light transmitted via scattering and absorption to a medium. A briefly description about it and related reference was added in the manuscript

*3.- The statement "Knowing that IgGs are bivalents, hypothetically, the ratio of binding site and antigen would be 4:1" is cryptic (to me). Do you mean that IgG bears 4 antigen-binding sites per molecule? Or that the molar ratio of IgG:antigen is 4:1? Please, revise and/or clarify!*

**Response:** As stated, the Ts5 was pre-incubated with 2 eqM of IgGs anti-Ts5. Knowing that IgGs are bivalents, in theory we should have 4 binding sites (two for each IgG) to Ts5. We detailed the sentence to avoid misunderstandings. Thanks to the referee to pointed out that.

*4.- "The same rate was used in the positive control group, using 0.5 ug of Ts5/animal paw pre-incubated 1 h at 37° C with 2 eqM of IgGs anti-Ts5". Please, specify the method used to quantify IgG. 0.5 ug Ts5 = 69.5 pmoles; 2eqM IgG = (2 x 69.5 x 10E-12) moles x 150000 g/mol = 20.85 ug. Is this correct?*

**Response:** As shown in the table 2, 0.5 μg of Ts5 corresponds to 33.5 pmoles (we considered the mass using epsilon). Two times the molarity of Ts5 molecules should be 67 pmoles of IgG, which corresponds to 10.05 μg. The mass of the IgG used was added in the manuscript.

*5.- Table 2. Please clarify the meaning of the two columns for "ug" (mass and e). If I understood it correctly the first column should be Absorbance units and the second column (A/e = concentration of toxin in mg/mL). However, the fact that Figure 4 refers to using 0.5 ug in the nociception assays induce to confusion! Similarly, in M&M: "For spontaneous nociceptive test, animals (n = 6) were challenged with intraplantar injection of TsV 95 or its isolated toxins (Ts1, Ts5, Ts6, Ts8, and Ts19 frag II) using 0.5 μg/paw (right hind paw)". Third column correspond to umoles of toxins which when dissolved in 10 uL give the listed molarity (uM). These figures are nowhere used in the manuscript; why are they listed in Table 2; All this should be better explained in the text and the columns headers should be labelled accordingly.*

**Response:** We appreciated the referee suggestion. The table 2 was reformulated accordingly. Only the values used in the study were kept. Regarding the assays, the stimuli were performed indeed with 0.5 ug of toxins (when they were estimated at 280 nm); however, the group received some critics regarding that, so the corresponding mass considering the epsilon was calculated later and detailed in the Table 2.

*6.- "The in silico analysis with Ts5 toxin demonstrated 4 predicted epitopes (Fig. 5B), that could be responsible to antibody binding (Fig. 5C)". Demonstrated? You may mean "suggested". The server is able to predict continuous B-cell epitopes with 65.93% accuracy using recurrent neural network (Saha, S and Raghava G.P.S. (2006) Prediction of Continuous B-cell Epitopes in an Antigen Using Recurrent Neural Network. Proteins,65(1),40-48). On the other hand, The suggested four epitopes cover almost the whole sequence of Ts5. The conclusion from the in silico analysis should be that the linear sequence of Ts5 may harbor one or more B-cell epitopes. Any evidence that any of the 4 predicted epitopes is actually recognized by the anti-Ts5 antibodies?, i.e. can synthetic peptides mimicking the predicted epitopes compete with anti-Ts5 antibodies for Ts5 binding?*

**Response:** As recommended, the term “demonstrated” was changed for “suggested”. The *in silico* B-cell epitopes prediction methods are known to achieve highly significant predictive performances (65 is not too bad), suggesting that these tools to be a powerful asset in rational epitope discovery. Regarding Ts5, there isn’t any study with the here predicted epitopes, but in theory the answer is “yes” the linear sequence could be recognized and activate at least 4 different B cell clones. The big issue to test them separately (small synthetic peptides) could be that they will not activate an inflammatory response to activate MHC-II and an adaptative immunity, even if administered with adjuvants. This can be supported by our study, in which most of mice immunized with the whole Ts5 molecule and CFA, had no memory humoral response. Anyway, epitopes predictions (for B and T cells) are considered a good tool to epitope discovery and recognized for vaccine strategies.

***Reviewer 2:***

*The article addresses a relevant topic, given the reduced number of studies that effectively assess the role of isolated Tityus toxins in the context of nociception.*

*However, some conclusions are hasty based on the results found.*

**Response:** We thank the referee to recognize that the study is pioneer to demonstrate the nociception induced by isolated and different classes of *T. serrulatus* venom toxins.

*- Evaluate the choice of concentrations used to prove the effects of Ts5, considering the percentage of this toxin in the venom, as well as the total amount of venom injected in an accident.*

**Response:** We are aware of this kind of limitation of the study. Our main aim in the biggening of the study was to test the toxins separately and to compare the nociceptive effect. As shown in Figure 1, it was very surprising the hyperalgesia caused by Ts5 alone, since TsV is a so powerful cocktail of neurotoxins. We then question if Ts5 would be the main neurotoxin responsible for the scorpion sting pain. But how to abolish the Ts5 from the venom? We decided to neutralize it. Considered that the anti-Ts5 polyclonal antibody (2 eqM) were able to complete neutralize the 0,5 μg of Ts5 (33.5 pmoles), we kept the same amount to neutralize the venom. According to the venom recovery, only 2% of the TsV is Ts5, in the TsV an amount of 0,01 μg or less of Ts5 is expected (1.3 pmoles). Since we used 67 pmoles of anti-Ts5 IgG, we could be sure that the number of antibodies were more than sufficient to neutralize all Ts5 from TsV. It is hard to extrapolate to a scorpion envenoming in humans, but for the referee have an idea on scorpion sting can inject around 50-100 μg of protein, about 1-2 μg of Ts5.

*- The use of polyclonal antibodies does not seem appropriate to evaluate the participation of a single toxin (Ts5) in the context of nociception, considering the percentage of identity among different toxins present in T. serrulatus venom.*

**Response:** We agree that the polyclonal antibody could also cross-neutralize other TsV toxins as well. However, the pain behavior was kept with high intensity even with Ts5 neutralization. Other protocol design discussed by group would also present drawbacks and would also be dependent of the antibody. We thought to build an affinity column with the polyclonal antibody and to elute the venom. But we could also remove other neurotoxins. Unfortunately, it was very hard to immunize animals and to purify and recover IgGs, being impracticable to immobilize in a CNBr Activated Agarose column.

*- The results obtained from in silico analysis of epitope prediction do not contribute to the discussion of the results, since no validation with the indicated epitopes was performed (e.g. isolation by immunoaffinity of specific polyclonal antibodies and their characterization).*

**Response:** The *in silico* B-cell epitopes prediction methods are known to achieve highly significant predictive performances, suggesting that these tools to be a powerful asset in rational epitope discovery. Regarding Ts5, there isn’t any study with the here predicted epitopes. Thus, we would like to keep the epitopes predictions.

*-The identification of exclusive epitopes and their use as immunogens for the production of protective antibodies could validate the importance of the neutralization of this toxin, in the context of the venom.*

**Response:** The linear epitopes were predicted; however, the big issue will be test them separately (small synthetic peptides). Based on their size, they may not activate an inflammatory response to activate MHC-II and an adaptative immunity, even if administered with adjuvants. This can be supported by our study, in which most of mice immunized with the whole Ts5 molecule and CFA, had no memory humoral response. Nevertheless, the group intend to study deeply them with other epitopes from other scorpion toxins.

*-The use of specific monoclonal antibodies to Ts5 could also be useful to inhibit the activities of this toxin.*

**Response:** In the sense of developing an antivenom indeed it would be very interesting to do that. But the study focused mainly in understanding the hyperalgesia effect induced by *Tityus serrulatus* toxins. On the other hand, we agreed with the referee that this study shows that Ts5 should be one of the targets of antivenoms to neutralize pain during Ts scorpion sting.

*-The evaluation of neutralization should have been performed with different concentrations of antibodies (confirm dose dependency).*

**Response:** Unfortunately, we did not enough amount of toxins and antibodies for that. Moreover, the aim was not to prove the neutralizing effect of a mice-derived polyclonal antibody, instead we focused on understanding the nociceptive effect trigged by Ts toxins.

*- The literature cited deserves an update and citations need to be adjusted (eg reference 38)*

**Response:** The references were reviewed and updated.

*- The video alone, without the proper controls, does not provide any conclusions.*

**Response:** The intention was to illustrate the nociceptive pain behavior, that is why we add as a Supplementary material. The entire results are demonstrated in the Figure 1A and B.
